# Sturge-Weber syndrome coexisting with episodes of rhabdomyolysis

**DOI:** 10.1186/1471-2377-13-169

**Published:** 2013-11-11

**Authors:** Min Zhu, Xiaobin Li, Meihong Zhou, Hui Wan, Yuchen Wu, Daojun Hong

**Affiliations:** 1 Department of Neurology, The first affiliated hospital of Nanchang University, Yong Wai Zheng Street 17#, Nanchang 330006, P.R. China

**Keywords:** Sturge-Weber syndrome, Rhabdomyolysis, Port-wine stains, Lipid metabolic myopathy

## Abstract

**Background:**

Sturge-Weber syndrome is a congenital neurocutaneous disorder characterized by facial port-wine stain, leptomeningeal angioma, and neurological disorders. Sturge-Weber syndrome can coexist with other disorders in a few patients; however, muscular abnormalities have not been reported in patients with Sturge-Weber syndrome.

**Case presentation:**

A Chinese girl presented with extensive port-wine stains, congenital bilateral glaucoma, and leptomeningeal angiomatosis. The neurocutaneous symptoms were consistent with the diagnostic criteria of Sturge-Weber syndrome. Meanwhile, episodes of rhabdomyolysis were supported by the recurrent symptoms as follows: exercise intolerance, hyperCKmia, elevated serum myoglobin, and renal failure. Myopathological features and high level of blood long-chain acyl-carnitine indicated that episodes of rhabdomyolysis might be caused by lipid metabolic myopathy. Causative mutations were not found in the CPT2, ACADVL, and GNAQ gene.

**Conclusions:**

We report the first case that Sturge-Weber syndrome coexists with episodes of rhabdomyolysis associated with lipid metabolic myopathy.

## Background

Sturge-Weber syndrome (SWS), also known as encephalofacial angiomatosis, is a congenital neurocutaneous disorder characterized by facial port-wine stains, leptomeningeal angioma, oculopathy, and neurological problems including seizures, stroke-like episodes, mental retardation, migraine, and hemiparesis [1,2]. Somatic activating mutations in guanine nucleotide-binding protein G(q) subunit alpha (GNAQ) gene have been found to be associated with Sturge-Weber syndrome [3]. The gene encodes a guanine nucleotide protein that plays an important role in regulating various cellular effectors. Mutant proteins disrupt vascular development leading to the malformation of blood vessels [3]. Sturge-Weber syndrome can coexist with hypomelanosis of Ito [4], polycystic kidney disease [5], and central hypothyroidism in a few patients [6]; however, muscular disorders have not been reported in patients with Sturge-Weber syndrome. We describe a patient presenting with extensive nevus flammeus, glaucoma, focal cerebral atrophy, and episodes of rhabdomyolysis. It is the first case that Sturge-Weber syndrome and episodes of rhabdomyolysis are observed together.

## Case presentation

### Clinical descriptions

A Chinese girl showed port-wine stains in bilateral faces, neck, trunk and extremities at birth. She had not opened eyes after birth, and then congenital bilateral glaucoma was diagnosed. She began to walk at age 1, but she had difficulty in running. She complained of muscle soreness or myalgia in lower limbs after long walking or stair climbing at age 2, but the symptoms can be relieved after having a rest. At age 7, she suddenly had left limbs weakness, accompanied by headache, nausea, vomiting, and aphasia without disturbance of consciousness. The symptoms were gradually relieved after 1 week. At age 10, a similar clinical episode happened again. At this time when she was 14 years old, she complained of limb myalgia, especially in both lower limbs after spring hiking. On the next day, she presented with left hemiplegia and headache. On the third day, she had aphasia and more severe hemiplegia. Subsequently, her consciousness gradually declined to somnolent state. The parent denied her history of suffering from epilepsy or taking antiepileptic drugs.

**Figure 1:**
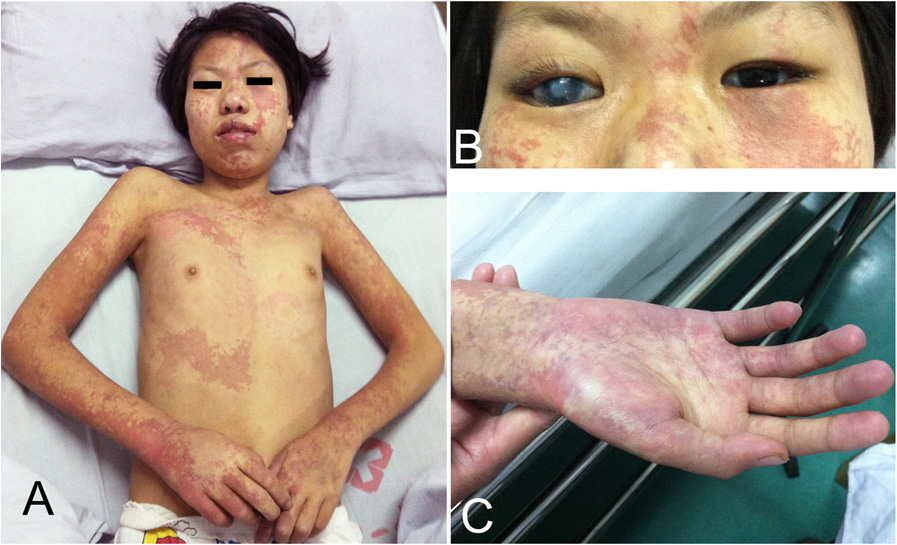
**Patient**’**s pictures.** Port-wine stains on bilateral faces, trunk and limbs **(A)**; right eye with buphthalmos and gray cornea **(B)**; port-wine stains in her hand **(C)**.

On admission, she was in comatose state, accompanied by episodes of twitching that began at left face, soon spread to left limbs. She had extensive port-wine stains distributing in both sides of her face, which also extended to her neck, trunk, and limbs (Figure 1A, 1C). The right eye was blind and exophthalmos (Figure 1B). The diameter of left pupil was 2.5*3 mm owing to crystal implantation at age 7. The left limbs had no response to pain stimulus. Babinski’s sign was positive on the left side.

**Figure 2:**
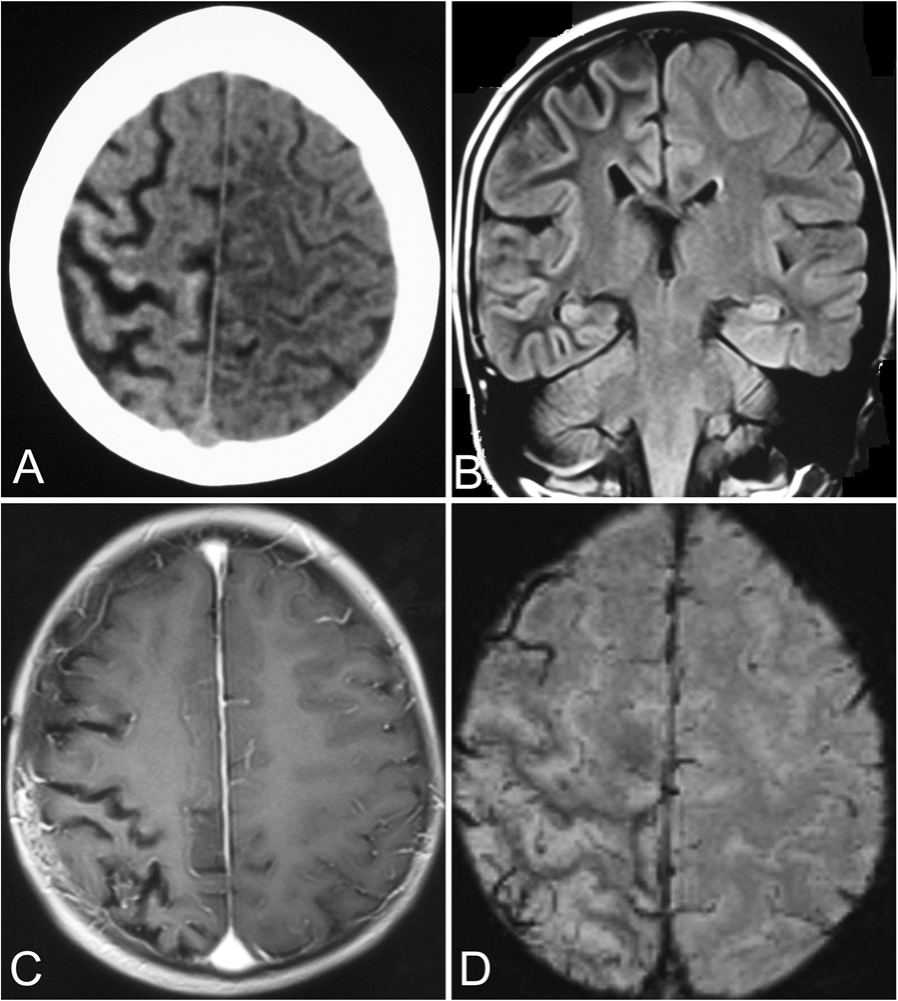
**Radiological characteristics of brain.** CT shows cortex atrophy and gyral calcifications in right parietal lobe **(A)**; coronal MRI shows the right side of the cerebral cortex atrophy **(B)**; enhanced MRI reveals mild leptomeningeal angiomatosis in right temporal-parietal areas **(C)**; SWI shows vein hyperplasia in the atrophic cerebral sulcus **(D)**.

Blood test showed serum creatinine 504 umol/L (44–106 umol/L), urea nitrogen 51.4 mmol/L (2.3-7.8 mmol/L), uric acid 1139 umol/L (150–440 umol/L), alanine aminotransferase 367 U/L (0–40 U/L), aspartate aminotransferase 670U/L (0–40 U/L), creatine kinase 44028 U/L (20–170 U/L), lactate dehydrogenase 1102 U/L (94–250 U/L), myoglobin 241 ug/L (50–85 ug/L). Urinary occult blood was strong positive (3+). Tandem mass spectrometry of blood revealed myristoyl carnitine (C14-1) 0.35 umol/L (0.02-0.25 umol/L), myristoyl-enoyl carnitine (C14:1–1) 0.83 umol/L, (0.012-0.3 umol/L), palmitoyl-enoyl carnitine (C16:1–1) 0.32 umol/L (0.02-0.2 umol/L). Blood routine, thyroid hormone and extractable nuclear antigen polypeptide spectrum were negative. Electroencephalogram revealed persistent δ activities on the right hemisphere. Cerebral CT showed focal atrophy of the right parietal lobe with gyral calcifications (Figure 2A). Cranial MRI revealed atrophy of cortex and other cerebral parenchyma in the right hemisphere (Figure 2B). Enhanced MRI showed mild leptomeningeal enhancement in right temporal-parietal areas (Figure 2C). Vein hyperplasia in atrophic cerebral sulcus was found on susceptibility weighted imaging (SWI) (Figure 2D).

**Figure 3:**
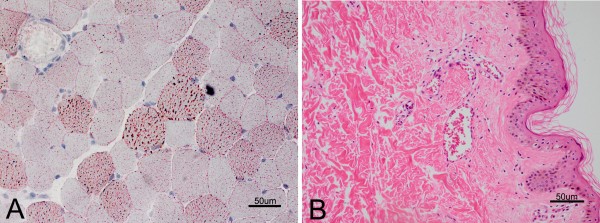
**Myopathological features.** lipid droplets accumulating in some fibers on oil red O stain **(A)**; skin biopsy reveals telangiectasias under epidermis on hematoxylin & eosin stain **(B)**.

After obtaining the parental consent, muscle biopsy revealed that some myofibers had numerous small round vacuoles, and absence of muscle fiber necrosis and regeneration. These vacuoles were filled with lipid droplets on oil red O (ORO) stain (Figure 3A). Skin biopsy revealed that the port-wine stains were constructed by many telangiectasias under epidermis (Figure 3B). The analysis of chromosome karyotype was normal. No mutation was found in the carnitine palmitoyltransferase-II (CPT2), very long-chain acyl-CoA dehydrogenase (ACADVL), and GNAQ gene in blood and muscle samples.

The patient was given antiepileptic drugs (carbamazepine 200 mg, bid; and phenobarbitol sodium 100 mg, im, q8h), multiple B vitamins and L-carnitine. The convulsion was completely controlled on the 6th day after admission. Consciousness was gradually improved and muscle strength of left limbs reached 3/5 grade (MRC grade), but she still had Broca’s aphasia. On the 6th day after admission, blood tests showed serum creatine kinase 20570 U/L, creatinine 108 umol/L, and urea nitrogen 12.7 mmol/L. On the 50th day after admission, port-wine stains were slightly faded and narrowed; muscle strength of left limbs was 4+/5 grade; aphasia was relieved; serum creatine kinase, urea nitrogen and creatinine were back to level of normal references. However, this girl experienced a similar clinical course again at 7 months after discharge.

## Discussion

Sturge-Weber syndrome is a rare congenital neurocutaneous disorder. The involved organs most often include brain, skin, eyes, and bones, while other organs are rarely involved [1,7]. Our patient presented with extensive port-wine stains, congenital bilateral glaucoma, stroke-like episodes, epilepsy, slight leptomeningeal angiomatosis, and cortical atrophy with gyral calcifications in the right cerebral hemisphere. The symptoms as described above can establish the diagnosis of Sturge-Weber syndrome [1,2]. Port-wine stains in patients with Sturge-Weber syndrome often distribute in the facial areas innervated by the first (V1) and second (V2) branches of trigeminal nerve, while the third (V3) branch and contralateral face can also be involved in some cases. In a few cases, the birthmark may spread to neck, chest, and upper limbs [1,8,9]. In the present case, port-wine stains were apparently extensive, not only in bilateral faces, but also in neck, trunk, and limbs. In addition, the leptomeningeal angioma was mild on enhanced MRI and SWI images, while the cortex atrophy of right hemisphere was apparent on MRI and CT images. To our best knowledge, this clinical phenotype is yet not to be reported in literatures [10].

On the other hand, the patient had extremely high level of serum CK, accompanied by increased myoglobin and acute renal failure. These symptoms indicated the diagnosis of rhabdomyolysis [11]. Rhabdomyolysis is a condition that damaged skeletal muscle fibers are broken down rapidly, and then the brokendown products (such as myoglobin) are released into blood. The muscle damage can result from physical factors, metabolisms, medications, infections, and hereditary disorders [11]. In this patient, muscle metabolic disorders may be the possible cause of rhabdomyolysis due to exercise intolerance and exercise-related muscle soreness since her childhood. Significant accumulation of lipid droplets in myofibers further supported that rhabdomyolysis may be related to lipid metabolic myopathy. Finally, the increased level of long-chain acyl-carnitine pointed to the possibility of carnitine palmitoyltransferase II deficiency [12] or very long chain acyl-CoA dehydrogenasedeficiency [13]. However, genetic analysis could not find causative mutations in the two genes. Exertional rhabdomyolysis caused by steal phenomenon of vascular malformation should be considered as the differential diagnosis [14,15]. In the present case, the numerous telangiectasias under epidermis (port-wine stains) might perform as steal phenomenon causing the muscle hypoperfusion that led to rhabdomyolysis. However, the results of myopathological changes and tandem mass spectrometry were not consistent with ischemic myopathy.

It has not been reported that the symptoms of Sturge-Weber syndrome and episodes of rhabdomyolysis simultaneously appeared in one patient. Some patients with Sturge-Weber syndrome can manifest as stroke-like episodes, which is easy to make a mistake with episodes of rhabdomyolysis. Therefore, it is important to bear in mind that Sturge-Weber syndrome can coexist with episodes of rhabdomyolysis.

Somatic GNAQ mutation can moderately activate the GPCR (G protein coupled receptors) downstream pathways as revealed in SWS patients [3]. Somatic GNAQ mutation can also result in blue nevi and melanocytic neoplasms of the central nervous system [16,17]. If the somatic mutation were to be presented in muscles, hyperactivation of the GPCR pathways could alter or impair muscle metabolisms that led to rhabdomyolysis. However, no GNAQ mutation is found in the muscle tissue of this patient. It seems that somatic GNAQ mutations are closely associated with dermatologic diseases. Neurocutaneous disorders usually involve germinal developmental abnormalities, including central nervous system, musculoskeletal system, eyes, and teeth [2]. Although muscular abnormalities have not been described in Sturge-Weber syndrome or Klippel-Trenaunay syndrome [18], both muscles and vessels are simultaneously developed from mesoderm. The common germinal origin lays the foundation for the possibility of Sturge-Weber syndrome coexisting with episodes of rhabdomyolysis. In addition, the rhabdomyolysis in this case may be related to genetic anomalies of lipid metabolism pathway. It is well known that gene mutations in association with lipid metabolism pathway can result in miscellaneous encephalopathy and ichthyosis [19]. Therefore, it is worthy to explore whether there is some intrinsic relationship between Sturge-Weber syndrome and episodes of rhabdomyolysis.

## Conclusions

We report the first case that Sturge-Weber syndrome coexists with episodes of rhabdomyolysis associated with lipid metabolic myopathy. Although the intrinsic relationship between Sturge-Weber syndrome and episodes of rhabdomyolysis is still unclear, the clinician should pay attention to differentiate stroke-like episodes of Sturge-Weber syndrome with episodes of rhabdomyolysis.

### Patient consent

Written informed consent was obtained from the patient’s parent for publication of this case report and any accompanying images. A copy of the written consent is available for review by the editor of this journal.

## Abbreviations

GNAQ: Guanine nucleotide-binding protein G(q) subunit alpha; CT: Computed tomography; MRI: Magnetic renounce image; SWI: Susceptibility weighted imaging; ORO: Oil red O; CK: Creatine kinase; CPT2: Carnitine palmitoyltransferase-II; ACADVL: Very long-chain acyl-CoA dehydrogenase; GPCR: G protein coupled receptors.

## Competing interests

The authors declare that they have no competing interests.

## Authors’ contributions

MZ, neurological evaluation of the patient and manuscript composition, HW and YW, neurological evaluation of the patient and suggestions regarding manuscript composition, XL and MZ, conduction of biospies and genetic analysis, DH, neurological evaluation of the patient, manuscript composition, corresponding author. All authors read and approved the final manuscript.

## Pre-publication history

The pre-publication history for this paper can be accessed here:

http://www.biomedcentral.com/1471-2377/13/169/prepub
